# Determination of traces of molybdenum and lead in foods by x-ray fluorescence spectrometry

**DOI:** 10.1186/2193-1801-3-341

**Published:** 2014-07-07

**Authors:** Muhammad Ali, Tasrina Rabia Choudhury, Babul Hossain, Md Panna Ali

**Affiliations:** Chemistry Division, Atomic Energy Centre, PO Box 164, Dhaka, 1000 Bangladesh; Department of Zoology, Rangpur Government College, Rangpur, Bangladesh; Entomology Division, Bangladesh Rice Research Institute (BRRI), Gazipur, 1701 Bangladesh

**Keywords:** EDXRF analysis, Food, Molybdenum, Lead, Matrix reduction, Carbonization

## Abstract

An Energy Dispersive X-ray Fluorescence (EDXRF) method using X-ray emitting isotopes in combination with pre-concentration by carbonization was developed to determine the levels of Mo and Pb accumulated in foods. The samples were carbonized at temperatures range of 150–400°C for 15 min to 2 h for powdering. The powder was then quickly formed into a pellet for EDXRF analysis. This analytical method (detection limit, 0.08 mg/kg) was used to determine levels of Mo and Pb in several kinds of foods from the local kitchen markets. The analytical results indicated that higher concentration of Mo (2.51 ± 0.09 mg/kg) and Pb (0.62 ± 0.13 mg/kg) was present in pulse. The maximum lead concentration is also found in pulses with the mean value of, which is far below the maximum permissible limit (ASP, pp 235, 1980) of Pb in food (1–5 mg/kg). The possibility of determination of traces of Mo and Pb in foods by x-ray fluorescence after carbonization is evaluated by comparative studies of standard reference materials. The method enables fast and direct analysis to be carried out without lengthy sample pretreatment and thereby minimizing sample contamination on a routine basis for food monitoring. No loss (<5%) of Mo and Pb is observed and a significant matrix reduction is achieved. Our findings highlighted that this method could be used for monitoring the levels of heavy metals (like Mo and Pb) accumulation in foods within short time and people can avoid health risk due to toxic effect of food.

## Introduction

Heavy metals are hazardous contaminants in food and the environment and non-biodegradable having long biological half-lives (Heidarieh et al. 2013). According to the World Health Organization (WHO 1995) heavy metals must be controlled in food sources in order to assure public health safety. Excessive concentration of food heavy metals is associated with the etiology of a number of diseases, especially cardiovascular, renal, neurological, and bone diseases (Chailapakul et al. 2007). A major reason to monitor levels of toxic metals in foods follows from the fact that contamination of the general environment has increased (Heidarieh et al. 2013). These metals could reach food chain through various biochemical process and ultimately biomagnified in various trophic levels and eventually threaten the health of human. Heavy metals are among the major contaminants of food supply and may considered the most important problem to our environment (Zaidi et al. 2005). Such problem is getting more serious all over the world especially in developing countries (Radwan and Salama 2006).

Due to increasing the health consciousness of the consumers and meet the food for rapid increasing population, sea food demands increase drastically during the past decades. Among the heavy metals, Lead (Pb) and cadmium (Cd) are significant environmental pollutants (Naser et al. 2009). Molybdenum is an essential trace element required by both plants and animals in very small amounts (Underwood 1977) and food is the major source for man (Tsongas et al. 1980). It is a component of several mammalian metalloenzymes including xanthine oxidase, aldehyde oxidase and sulfite oxidase. The relationship between molybdenum lack and dental caries, oesophageal carcinoma and molybdenum deficiency in humans has been confirmed (Davies 1977). There has been a possible relationship between a high intake of molybdenum and a high level of incidence of gout. The major toxic effects of lead have been described (NAS 1971); they include anaemia, neurological dysfunction, and renal impairment. Childhood lead poisoning is a major public health problem in many industrialized countries (Parsons and Slavin 1993) and is arguably one of the most preventable environmental diseases (Parsons et al. 1997). Thus, trace elements essential or toxic play an important role in human health and diseases, and their main pathway to reach the human body is the food chain. Therefore, to safeguard human health and to protect environment, it is very important to know the level of Mo and Pb in foods and their dietary intake. Hence, many analytical methods for rapid and routine determinations of Mo and Pb in a complex matrix like foods have been developed. Atomic Absorption and Inductively Coupled Plasma Atomic Emission Spectrometry (ICP-AES) are commonly used but these methods require time-consuming digestion procedures such as dry ashing or wet digestion. Dry ashing at higher temperatures may lead to losses of Mo and Pb by retention on the surfaces of reaction vessels at high temperatures for longer time, and the use of chemical reagents in wet digestion may cause contamination (Southgate 1987). Nevertheless, complete dry or wet digestion of food items and their dissolution is often troublesome and time consuming. Therefore, in the present research, attention has been given to the use of XRF with a sample pretreatment for carbonization but not for ash by dry ashing or dissolution by wet digestion. The dry ashing or wet digestion of foods is not usually satisfactory for XRF or PIXE measurements as both the methods have mostly been applied to analytical problems where sample materials have been used in solid forms rather than in ash or solution. It is often difficult to prepare pellets from ash without a suitable binder. Preparing thick pellets from carbonized and powdered food samples without employing a binder is an easy technique often used for the determination of elements in a sample by XRF or PIXE spectrometry with better sensitivity. The detection limit of the XRF method is comparatively higher in solutions than in solid homogeneous fine samples (Kalam et al. 1978). The solid sample in a pellet form is suitable for PIXE or XRF as thick targets are generally less complicated and faster to prepare than thin targets, and involve less risk of contamination and loss of elements to be analyzed. A comparative study of the sensitivity of XRF and PIXE methods for trace element analysis in thick solid biological targets have been made by Ali (1995). From that study it was observed that photon beam excites a wider range of elements and produce better sensitivity for higher Z elements (Mo and Pb) than PIXE. These two elements having relatively higher Z values have higher x-ray production cross-sections with gamma photon interactions. However, the technique suffers from serious matrix effects, and hence a special methodology has to be developed for each matrix type, if quantitative analytical data are requested (Sieghbahn 1965; Delgado et al. 1987; Dos Santos and Conde 1987). Therefore, EDXRF has been applied for the determination of Mo and Pb in foods without any tedious pretreatment procedures, usual in the analysis of biological samples. A serious drawback of the XRF method is the rather complicated way of quantitative evaluation of the matrix effects. Based on comparison of the spectra of samples with those of closely matched standard reference materials, a relatively simple method has been worked out for quantitative determinations on a routine basis, which, besides minimizing the errors, fits into the system of the classical methods of quantitative analysis.

## Experimental methology

### Sampling and sample preparation

Food samples were purchased from local kitchen markets, carried to the laboratory and stored in plastic Ziploc bags in a refrigerator. All samples were thoroughly cleaned first under tap water and then finally with distilled de-ionized water, peeled, shelled, or deboned as applicable, and the edible portion was taken for analysis. The edible portion of each cleaned samples (100 g) were first air dried and then oven dried in an electric oven at 85 ± 5°C to a constant weight to get dry weight factor before carbonization and then carbonized in a muffle furnace in the range of 150–400°C in pre-weighed porcelain dishes for 15 min. to 2 h according to sample type. Comparison of the samples carbonized at temperatures of 150–400°C showed that treatment at 300°C for 1–2 h provides good carbonization conditions and this was applied for subsequent carbonization of most of the plant foods. But most of the animal foods were carbonized at 400°C. The optimum temperature for carbonization of cereals was 400 and 200°C for vegetables and the time required for this treatment was 1 h. Initially, the samples were dried and then heated, and the process of carbonization to ashing was observed. The results showed that heating of the plant foods at 350°C for 15 min. to 30 min. and animal foods at 550°C for more than 1 h start ashing. The standard reference materials, bovine liver (NIST-SRM-1577b), horse kidney (IAEA-H-8), animal bone (IAEA-H-5), lobster hepatopancreas (NRCC-TORT-2), rice flour (NIST-SRM-1568a), pine needles (NIST-SRM-1575), orchard leaves (NIST-SRM-1571), and tomato leaves (NIST-SRM-1573) were treated as the samples and analyzed to evaluate the loss of Mo and Pb during sample treatment. Both the carbonized standards and samples were powdered and homogenized in an aluminum carbide mortar. The homogenized fine powders were then pressed into pellets of 100 mg weight, 10 mm diameter by a hand press pellet maker. Geometric and measurement parameters were the same for each standard and sample.

### Analytical method

A radioisotope-induced X-ray fluorescence system was applied for EDXRF analysis, where a 30-mCi Cd-109 annular x-ray source was used for excitation. The components of an energy-dispersive x-ray spectrometer developed in this laboratory consist of a primary x-ray source, sample holder, an x-ray detector, a multi-channel analyzer (MCA) and associated NIM electronics for data acquisition and processing. A microcomputer is dedicated to this system for on-line XRF data analysis.

**Figure 1:**
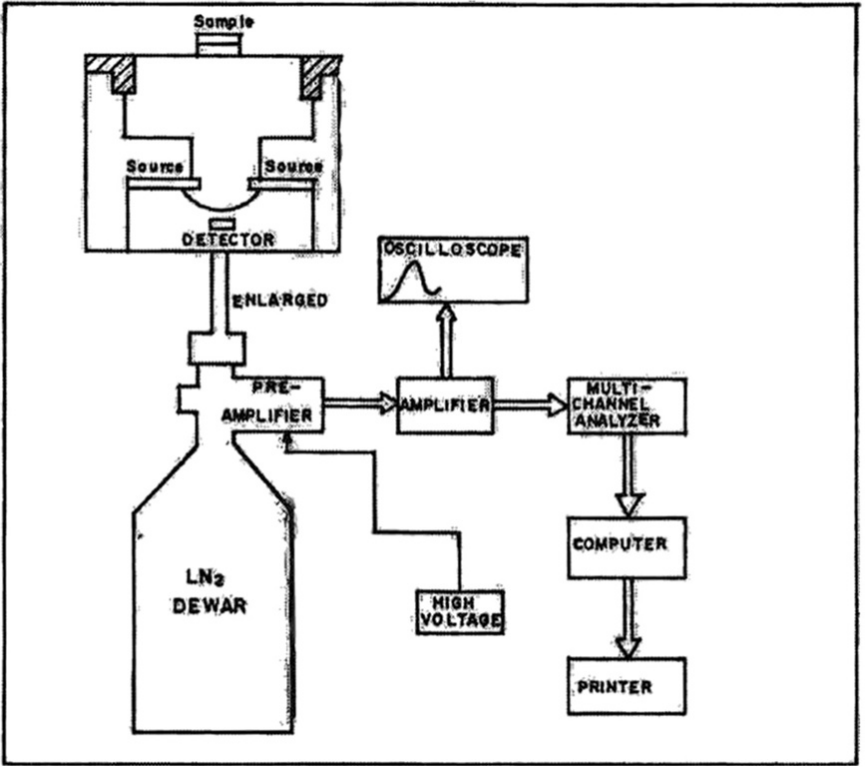
**The schematic diagram of the radioisotope – induced XRF method.**

The source of primary x-rays for excitation of characteristic x-rays is a high intensity ^109^Cd annular sealed x-ray source. The sample holder is simply a receptacle to hold the sample. The excitation source is a ring type construction, which is ideal for the Si(Li) detector. The sample is directly irradiated from the source. The source holder is made of pure aluminum and different sample holders can be used for different samples depending on their forms, shapes and sizes. The overall experimental setup for this system is illustrated in Figure 1. In the present setup, the relative distance of source from the detector and source from the sample can be changed to obtain a desired optimum source-sample-detector geometry condition for analysis. This setup also provides an easier sample handling provision. Since the setup is operated in open air, the spectral background is relatively low and the sensitivity is relatively high, relatively for higher elements. By this experimental setup the K-lines from molybdenum and L-lines from lead can be measured precisely. The targets were placed above the Cd source, which was positioned vertically over the detector as shown in Figure 1.

**Figure 2:**
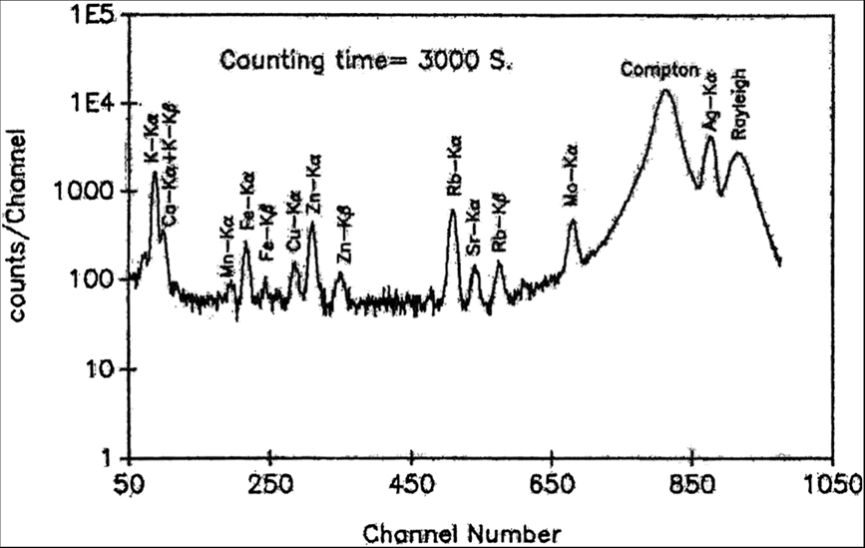
**A typical XRF spectrum obtained from a carbonized pulse sample.**

The characteristic x-rays were detected with an Ortec Si(Li) detector. The resolution of the detector was 175 eV at 5.9 keV. The samples were excited for 3000 seconds in the form of pellets and the peak used for analysis was Kα for Mo and Lα for Pb. The characteristic X-rays were detected with a Si(Li) Ortec detector and analyzed with a 1024 multichannel analyzer (Canberra) and other NIM electronics. A typical EDXRF spectrum obtained from a carbonized green gram sample under these experimental conditions is illustrated in Figure 2. All peak areas were integrated using the FORTRAN software AXIL (analysis of x-ray spectra by iterative least squares program) on a Pentium II (IBM compatible) professional computer. The tailored version of the software is PRO/QXAS provided by the International Atomic Energy Agency (IAEA), Vienna, Austria (Vekemans et al. 1994).

## Results and discussion

### Concentration calibration

**Figure 3:**
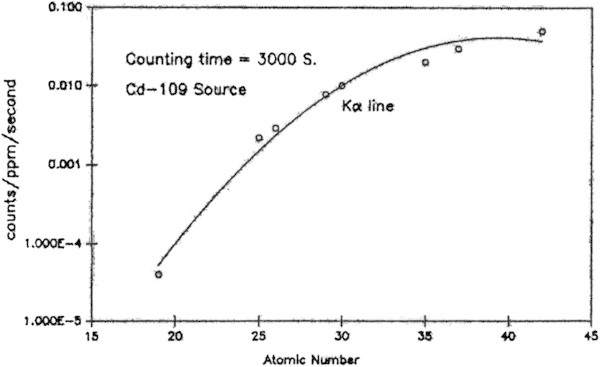
**The X-ray yield curve for concentration calibration constructed from the NIST bovine liver standard, SRM 1577b.**

In the case of XRF analysis, the concentration calibration is defined as the number of x-rays per ppm of the element of interest per second vs. its atomic number. The concentrations of Mo and Pb in foodstuffs were calculated by comparison with the calibration curves constructed from the standards, bovine liver (NIST-SRM-1577b) and orchard leaves (NIST-SRM-1571. The concentration calibration curves were constructed from the average peak areas obtained from the irradiation of five 100 mg standard pellets prepared in the same way as the sample pellets. A typical calibration curve obtained from the bovine liver standard for 3000 s irradiation with 30 mCi, Cd source is given in Figure 3. Using the calibration curves, the concentration of an element ‘e’ in ppm (μg/g) in a given sample is obtained from the following expression:

1

Where, C_e_ = concentration of the element ‘e’ in the sample in ppm; Ye = X-ray yield of the element ‘e’ in the sample in counts/second; S_e_ = Calibration factor (sensitivity) of the element ‘e’ in counts/ppm/second; D = Ratio of wet weight to the dry weight of the sample and  = Conversion factor of the estimated concentration from dry state of the sample to the wet state.

### Matrix effect

In trace analysis, the ideal situation is to use a standard with the same matrix composition as the sample to minimize matrix effects. Such a situation is not always possible to achieve because of the non availability of the standard with the same matrix composition as the sample particularly for different varieties of food samples. But as an alternative, it is suitable to use a standard with the bulk of the matrix having a light element composition (H, C, N, O, S), which is similar to the sample matrix. For the proper application of XRF method, the standard reference materials are chosen those matches as closely as possible, with respect to matrix type and concentration of the measurands of interest, the food samples those are to be analyzed. In this study, the use of the bovine liver and orchard leaves standards for Mo and Pb analysis in foods is justified by the fact that both the samples and the standards have a predominantly light element biological matrix and therefore can be assumed to have an almost similar matrix composition. This assumption was experimentally investigated (Ali 1995; Biswas et al. 1984) and it was observed that the systematic deviation in the calibration due to different matrices such as orchard leaves, pine needles, bovine liver, animal bone, horse kidney, lobster hepatopancreas etc. was estimated to be less than 10% for most of the elements. Sample homogeneity was tested by replicate irradiations and was found to be within 5%. The above discussions validate the calibration procedures used in this study.

### Evaluation of the loss of elements

The dry destruction of organic matter is an accepted procedure if the element is of low volatility. The optimum carbonization technique was applied here because molybdenum is a refractory element and both the molybdenum and lead do not present problems of volatility. The standard reference materials, bovine liver (NIST-SRM-1577b), horse kidney (IAEA-H-8), animal bone (IAEA-H-5), lobster hepatopancreas (NRCC-TORT-2), rice flour (NIST-SRM-1568a), pine needles (NIST-SRM-1575), orchard leaves (NIST-SRM-1571), and tomato leaves (NIST-SRM-1573) were treated as the samples and analyzed to evaluate the loss of Mo and Pb during sample treatment. The analytical results of Mo and Pb in different biological standards after carbonization are presented in Tables 1 and 2 respectively. These experimental values indicate that the measurement accuracy of the method, varied over the range of 2–11%. The values agreed well with the certified values under the present experimental conditions, which proved that the XRF system described here can be applied to the determination of Mo and Pb in food samples. On the other hand, it also enabled the method based on comparison with reference standards to be applied to biological samples.

**Table 1:** **Analytical results of molybdenum in different biological standards heated at 400°C for 1 h**

Reference Material	Concentration in mg/kg		Accuracy (%)
	Measured value (N = 5)	Certified value	
Bovine liver	3.30 (±0.41)	3.5 (±0.30)	5.7
Rice flour	1.50 (±0.15)	1.46 (±0.10)	2.7
Horse kidney	2.30 (±0.28)	2.40 (±0.30)	4.2
Lobster Hepatopancreas	0.86 (±0.20)	0.95 (±0.10)	9.5

N = Number of measurements. Uncertainties are counting statistics.

**Table 2:** **Analytical results of lead in different biological standards heated at 400°C for 1 h**

Reference Material	Concentration in mg/kg		Accuracy (%)
	Measured value (N = 5)	Certified value	
Animal bone	3.20 (±0.50)	3.10 (±0.60)	3.2
Orchard leaves	43.0 (±4.00)	45.0 (±3.00)	4.4
Pine needles	11.50 (±0.70)	10.80 (±0.50)	6.5
Lobster	0.31 (±0.15)	0.35 (±0.13)	11.4
hepatopancreas Tomato leaves	5.80 (±0.30)	6.30 (±0.30)	7.9

N = Number of measurements. Uncertainties are counting statistics.

### Application

The method was applied to various food types selected to cover a wide range of composition and consumption, with representative lipid, protein and glucoside contents. The selected foods were cereals, pulses, vegetables, meats, fishes, milk and milk products, eggs, fruits etc. The results obtained in different groups of food are given in Table 3. It is found from Table 3 that some of our food values for Mo are comparable and close to those for US food values (Vazquez-Gonzalez et al. 1989). These are for our wheat flour is (0.31 ± 0.05) mg/kg and US wheat flour is (0.26 ± 0.09) mg/kg; for our lentil is (2.83 ± 0.10) mg/kg and US lentil is (3.86 ± 0.229) mg/kg; for our beef is (0.10 ± 0.02) mg/kg and US beef is (0.11 ± 0.009) mg/kg; for our hen egg is (0.20 ± 0.03) mg/kg and US egg is (0.20 ± 0.18) mg/kg and for apple in Bangladesh is (0.26 ± 0.02) mg/kg and for apple in US is (0.20 ± 0.018) mg/kg. Some of our pulse lead values are also comparable and close to those of Pakistani pulse values. The values are 0.55 ± 0.02, 0.45 ± 0.12, and 0.74 ± 0.18 mg/kg in Bangladeshi pulses where as the corresponding values in Pakistani pulses (Qureshi et al. 1990) are 0.53 ± 0.02, 0.51 ± 0.03, and 0.73 ± 0.02 mg/kg respectively. In order to demonstrate the validity of the EDXRF data in Table 3, measurements of Mo and Pb in some food groups after carbonization are also performed using proton induced x-ray emission (PIXE) spectrometry and the results are found to be in good agreement with the EDXRF data.

**Table 3:** **Representative values of molybdenum and lead concentrations in different food groups (in mg/kg, fresh weight)**

Food group	Mo	Pb
***Cereals (mean)***	1.50 ± 0.06	0.57 ± 0.02
Rice, (IRRI, N = 15)	4.44 ± 0.11	0.68 ± 0.01
	3.98 ± 0.10*	0.72 ± 0.02*
Rice (local, N = 12)	0.75 ± 0.01	0.82 ± 0.01
	0.65 ± 0.02*	0.75 ± 0.02*
Rice sunned (milled, N = 10)	0.49 ± 0.08	0.65 ± 0.01
	0.54 ± 0.09*	0.70 ± 0.03*
Wheat flour (coarse, N = 5)	0.31 ± 0.05	0.11 ± 0.04
	0.26 ± 0.09**	
***Pulses (mean)***	2.51 ± 0.09	0.62 ± 0.13
Bengal gram (split, N = 3)	0.77 ± 0.08	0.55 ± 0.02
		0.53 ± 0.02***
Black gram (split, N = 3)	2.85 ± 0.07	0.45 ± 0.12
		0.51 ± 0.03***
	2.78 ± 0.08*	0.46 ± 0.15*
Green gram (split, N = 3)	3.18 ± 0.07	0.45 ± 0.11
Lentils (N = 3)	2.83 ± 0.10	0.74 ± 0.18
	3.86**	0.73 ± 0.02***
Lathyrus pea (N = 3)	2.93 ± 0.12	0.92 ± 0.24
***Vegetables (mean)***	0.08 ± 0.03	0.20 ± 0.04
Vegetables (green, N = 15)	0.09 **±** 0.04	0.22 **±** 0.05
Potatoes (N = 3)	0.06 ± 0.02	0.25 ± 0.04
	0.07 ± 0.004**	
Tomatoes (N = 3)	0.05 ± 0.02	0.12 ± 0.03
Cabbage (N = 3)	0.10 ± 0.03	0.15 ± 0.05
***Meats (mean)***	0.07 ± 0.01	0.59 ± 0.05
Chicken (N = 5)	0.06 ± 0.01	0.67 ± 0.04
Goat (N = 5)	0.05 ± 0.01	0.25 ± 0.04
Beef (N = 5)	0.10 ± 0.02	0.85 ± 0.08
	0.11 ± 0.009**	
***Fishes (mean)***	0.12 ± 0.03	0.14 ± 0.07
Shrimp (N = 3)	0.09 ± 0.02	0.15 ± 0.08
Prawn (N = 3)	0.12 ± 0.02	0.13 ± 0.07
Fish (sweet water, N = 15)	0.12 ± 0.03	0.15 ± 0.06
Fish (marine, N = 6)	0.13 ± 0.04	0.11 ± 0.05
	0.15 ± 0.06*	0.13 ± 0.04*
***Milk and milk products (mean)***	0.26 ± 0.06	
Redcow (N = 3)	0.35 ± 0.09	< 0.08
Dano (N = 3)	0.31 ± 0.05	< 0.08
Cheese (N = 3)	0.11 ± 0.04	< 0.08
***Eggs (mean)***	0.18 ± 0.03	0.16 ± 0.06
Duck egg (N = 10)	0.15 ± 0.02	0.17 ± 0.06
Hen egg (N = 10)	0.20 ± 0.03	0.15 ± 0.05
	0.23 ± 0.009**	
***Fruits (mean)***	0.13 ± 0.01	0.23 ± 0.03
Orange (N = 3)	0.13 ± 0.02	0.19 ± 0.01
Mango (N = 3)	0.09 ± 0.01	0.11 ± 0.02
Pineapple (N = 3)	0.10 ± 0.01	0.11 ± 0.01
Banana (N = 3)	0.09 ± 0.01	0.40 ± 0.07
Jackfruit (N = 3)	0.13 ± 0.01	0.44 ± 0.06
Apple (N = 3)	0.26 ± 0.02	0.11 ± 0.03
	0.20 ± 0.018**	
***Tea*** *(N = 3)*	0.34 ± 0.09	3.38 ± 0.29

N = Number of samples. Uncertainties are due to the counting statistics.

*Values are measured by PIXE. **Values are taken from (Vazquez-Gonzalez et al. 1989).

***Values are taken from (Qureshi et al. 1990).

The mean and range of Mo and Pb contents of different food classes repeatedly sampled in this study are given in Tables 4 and 5 respectively. It is found that there are considerable variations in the content of molybdenum and lead from group to group not only in Bangladeshi foods but also in Australian (Fardy et al. 1994) and US foods and this can be explained mainly in terms of their availability in the local environment. The concentration of molybdenum in all food groups studied in Bangladesh is found to be maximum in pulses with the range of 0.77–3.18 mg/kg and the mean of 2.51 ± 0.09 mg/kg. The cereal contents 1.50 ± 0.06 mg/kg of Mo, which is comparable to pulse value. But the other food groups are poor source of Mo. The distribution pattern of Mo contents in different food varieties is found to follow the order: pulses > cereals > milk and milk products > eggs > fruits > fishes > vegetables > meats. The maximum lead concentration is also found in pulses with the mean value of 0.62 ± 0.13 mg/kg, which is far below the maximum permissible limit (Reilly 1980) of Pb in food (1–5 mg/kg). But this pattern is no longer true in case of other foods. The Pb contents in different food classes follow pulses > meats > cereals > fruits > vegetables > eggs > fishes > milk and milk products and the corresponding mean values are: 0.62, 0.59, 0.57, 0.23, 0.20, 0.16, 0.14 and < 0.08 mg/kg. The Australian grain products also contain the highest range of Pb among food classes but this range value (0.01–1.80) mg/kg is much higher than our range value of (0.11–0.82) mg/kg. The Pb concentrations found in different Bangladeshi food classes revealed that these are not contaminated with lead through the environmental or ecological imbalance of this element.

**Table 4:** **Molybdenum concentration in different food groups in mg/kg, fresh weight basis**

Food group		Bangladeshi food	Australian food (Fardy et al. 1994)	US food (Vazquez-Gonzalez et al. 1989)
Cereals:	Range	0.31–4.44	0.06–0.64	0.14–1.80
	Mean	1.50 ± 0.06	-	0.57
Pulses:	Range	0.77–3.18	-	-
	Mean	2.51 ± 0.09	-	3.30
Vegetables:	Range	0.05–0.10	0.01–0.20	0.01–0.08
	Mean	0.08 ± 0.03	-	0.05
Meats:	Range	0.05–0.10	0.01–0.25	0.02–0.05
	Mean	0.07 ± 0.01	-	0.04
Fishes:	Range	0.09–0.13	0.20–0.54	0.01–0.02
	Mean	0.12 ± 0.03	-	0.01
Milks:	Range	0.11–0.35	0.02–0.04	0.05–0.11
	Mean	0.26 ± 0.06	-	0.07
Eggs:	Range	0.15–0.20	0.12–1.58	-
	Mean	0.18 ± 0.03	-	0.09
Fruits:	Range	0.09–0.26	0.01–0.03	0.01–0.09
	Mean	0.13 ± 0.01	-	0.03

Uncertainties are due to the counting statistics.

**Table 5:** **Lead concentration in different food groups in mg/kg, fresh weight basis**

Food group		Bangladeshi food	Australian food (Fardy et al. 1994)	Australian Legal Maximum
Cereals:	Range	0.11–0.82	0.01–1.80	-
	Mean	0.57 ± 0.02	-	2.5
Pulses:	Range	0.45–0.92	-	-
	Mean	0.62 ± 0.13	-	-
Vegetables:	Range	0.12–0.25	0.01–0.14	-
	Mean	0.20 ± 0.04	-	2.0
Meats:	Range	0.25–0.85	0.70–0.78	-
	Mean	0.59 ± 0.05	-	1.5
Fishes:	Range	0.11–0.15	0.04–0.10	-
	Mean	0.14 ± 0.07	-	2.5
Milks:	Range	-	0.01–0.04	-
	Mean	-	-	1.5
Eggs:	Range	0.15–0.17	0.07–1.50	-
	Mean	0.16 ± 0.06	-	1.5
Fruits:	Range	0.11–0.44	0.02–0.20	-
	Mean	0.23 ± 0.03	-	1.5

Uncertainties are due to the counting statistics.

The present findings of Mo, however, indicate that the nutritional value in terms of its contents in Bangladeshi common foods are at least comparable with those reported in other countries (Vazquez-Gonzalez et al. 1989; Fardy et al. 1994) The present carbonization technique is, with a few exceptions, the most suitable method (Table 3). This technique is found suitable for samples of all types except fatty fish and fats and oils. This dry carbonization method enhances the detection limit from a few ppm levels to a few ppb levels by reducing the organic mass from 30–90 folds for fresh foods. The minimum detection limit (MDL) of the present method is matrix dependent and for Mo and Pb determinations in a plant matrix (pine needles, dry weight) are 0.02 and 0.08 respectively. This detection limit can still be lowered by increasing the irradiation time. It is suitable for the analysis of Mo and Pb in a large number food samples for food monitoring in a short time.

### Dietary intake and health effects

The values of dietary intake of Mo and Pb are still scarce in the literature reports in developing countries (Parr et al. 1992) and this is very important information required in assessing risks to human health due to their deficiency or overburden. In assessing this risk, knowledge of the current levels of dietary intake of population groups are of primary importance. For Bangladesh, hitherto no such dietary intake data are available yet. Therefore, the daily dietary intake of Mo and Pb for different population groups in Bangladesh has been calculated.

The total daily dietary intake quantity of Mo and Pb from foods were calculated by multiplying the mean concentration of the respective element measured and calculated in a food group in the present work by the weight of that group consumed (Ali 1995) by each population group, and then summing these products for all food groups. The consumption multiplied by the concentration yields the intake. The daily intake of an element (Ei) could then be calculated by using the equation, Ei = ∑FiCi; Where, Fi is the weighted daily intake of food item i (g/person/day) and Ci is the element content in the relevant food group i (μg/g, fresh weight).

Using the above-mentioned equation, the daily Mo and Pb intake by different population groups such as Children, Adolescent, Adult Male (AM), Adult Femele (AF), Pregnant women (PW) and Lactating Women (LW) in Bangladesh (BD) are estimated.

The calculated total daily intakes are compared with the Recommended Daily Intakes (RDI), set by the United States Food and Nutrition Board and intakes recently published for other countries (Parr et al. 1992). The present results obtained from dietary intake of Mo and Pb, their distribution in various population groups and the importance of different food group’s contributions in total daily intake are discussed as follows:

The calculated total daily intakes of Mo by different population groups are 0.45 mg for children, 0.86 mg for adolescent, 1.16 mg for adult male, 0.83 mg for adult female, 0.82 mg for pregnant women and 0.92 mg for lactating women.

The Mo intakes by all age and sex groups compared to the US FNB recommended intake values are found higher than the safe levels of 0.30 mg for children and 0.50 mg for other population groups (NAS 1989). Although only a few reports have been published on molybdenum toxicity in humans, there is an excessive exposure, at present, to this element instead of having deficiency in human. An extremely high incidence of gout in some areas of Armenia, USSR, among communities subsisting mainly on locally grown foods, has been associated with abnormally high concentrations of Mo in soil and plants (Kovalsky et al. 1961). The Mo intake of individuals in affected areas was 10–15 mg/day compared to 1–2 mg in nearby areas where the population incidence of gout was low. Absorption of Mo from the gastro-intestinal tract is high. In humans about 50 of ingested Mo enters into the blood stream. The Bangladesh Mo intake ranges from 0.45–1.16 mg/day, which would be possibly a cause of low incidence of gout in our population. Supporting other studies is needed to confirm this incidence. The higher intakes of Mo by Bangladeshi population groups as revealed in this study may be attributed to the major contribution of some specific foods such as cereals and pulses in the traditional Bangladeshi diets. About 90% of the Bangladeshi diets are composed of cereals, pulses and vegetables.

The total daily Pb intake data of Bangladeshi population has been estimated and these are 188 μg for children, 355 μg for adolescent, 482 μg for adult male, 339 μg for pregnant women and 383 μg for lactating women. With a very few exceptions, Pb intakes in Bangladeshi population groups (age and sex) are comparable to the values reported to many other countries (Parr et al. 1992). In the USA, lead intake from foods range from 200–300 μg per day (NAS 1972) but values in excess of 400 μg of lead per day have been reported from western Europe (WHO 1973). The Bangladeshi adult intake (sexes combined) is 411 μg, which is higher than the western European value (400 μg) but lower than 514 μg that was found in one Indian investigation (Jather et al. 1981). according to Clarkson (Reilly 1980) the maximum permissible daily total intake of Pb for adult should be 800 μg. Compared to this value; the Bangladeshi intake is lower and is only 51% of the maximum permissible daily total intake (800 μg). The present intake of Pb (411 μg) by the adult population of Bangladesh is also below the provisional tolerable daily intake of 429 μg/day (WHO 1978).

From the results of this dietary analysis, it is observed that the major foodstuff contributors to daily dietary intakes of Mo and Pb are plant foods (cereals and pulses) while the contribution of animal foods (meats, fishes, milk milk products, and eggs) is negligible. The main food of the Bangladeshi population is rice or wheat followed by pulses or vegetables and hence concentrations of Mo and Pb in cereals contribute a major part to the total daily intake of these elements. Although the mean concentration of Mo and Pb is higher in pulses, the consumption fraction of this ingredient is comparatively low. The daily consumption of other food classes such as meats, fishes, milks, eggs and fruits are generally much less compared to cereals and pulses and hence their contribution to the total daily intake of Mo and Pb is quite low.

Different groups of food items such as cereals, pulses, vegetables, meats, fishes, milks, eggs and fruits, commonly consumed in Bangladesh, have been studied by XRF method for low levels of Mo and Pb generally present in food matrix. The samples were analyzed in the form of thick pellets. To improve the sensitivity of the method, in all the analyses, samples were carbonized under appropriate conditions. This dry carbonization method is rapid, simple, low cost, uses no reagents and requires low temperatures and almost free from contamination is comparison with dry and wet ashing. The significant conclusions those can be drawn from the dietary analysis are: (i) With a few exceptions, the Bangladeshi food values in terms of Mo and Pb contents are comparable to those from other countries and are not heavily burdened with toxic element such as Pb. (ii) The findings of the dietary intake study suggest that much of the Mo and Pb intake has come from cereals, pulses and vegetables and (iii) The daily dietary intake level of Pb for adult Bangladeshi population is low in comparison with recommended safe intake and are comparable to those in many other countries. This is very significant in view of the fact that Bangladesh is at the low level of industrialization.

In summary, to determine heavy metal levels in foodstuffs, we developed a relatively simple and reliable XRF (using x-ray emitting isotopes) in which the sample is only homogenized, dry carbonized and formed into a pellet for X-ray measurements. This method could be applied for a wide range of food (both in plant and animal) in a low level of contaminants.

### Compliance with ethics requirements

**For articles that do not contain studies with human or animal subjects:**

This article does not contain any studies with human or animal subjects.
